# Utility of next-generation sequencing in genetic testing and counseling of disorders involving the musculoskeletal system—trends observed from a single genetic unit

**DOI:** 10.1186/s13018-022-02969-x

**Published:** 2022-02-05

**Authors:** Gayatri R. Iyer, Roshan Kumar, Subhadra Poornima, Aruna Priya Kamireddy, Keerthi Konda Juturu, Lekhangda Bhatnagar, Srinka Arora, Vaishnavi Suresh, Prashant R. Utage, Sarah Bailur, Akhilesh N. Pujar, Qurratulain Hasan

**Affiliations:** 1 Department of Genetics and Molecular Medicine, Kamineni Hospitals, LB Nagar, Hyderabad, Telangana 500 074 India; 2 Department of Orthopedics, Kamineni Hospitals, LB Nagar, Hyderabad, India; 3 Department of Genetics, Credence Diagnostic Centre, Moula Ali, Hyderabad, India; 4 Utage Child Neuro Clinic and Development Centre, Narayanguda, Hyderabad, India; 5 Department of Pediatrics, Kamineni Hospitals, LB Nagar, Hyderabad, India; 6 Department of Ocular Genetics, LV Prasad Eye Institute, Banjara Hills, Hyderabad, India

**Keywords:** Musculoskeletal disorders, Skeletal dysplasia, Next-generation sequencing, Genetic counseling, Molecular diagnosis

## Abstract

**Background:**

Disorders involving the musculoskeletal system are often identified with short stature and a range of orthopedic problems. The clinical and genetic heterogeneity of these diseases along with several characteristic overlaps makes definitive diagnosis difficult for clinicians. Hence, using molecular testing in addition to conventional tests becomes essential for appropriate diagnosis and management.

**Methods:**

Comprehensive clinical examination, detailed pretest and posttest counseling, molecular diagnosis with next-generation sequencing (NGS), genotype–phenotype correlation and Sanger sequencing for targeted variant analysis.

**Results:**

This manuscript reports a molecular spectrum of variants in 34 orthopedic cases referred to a single genetic unit attached to a tertiary care hospital. The diagnostic yield of NGS-based tests coupled with genetic counseling and segregation analysis was 79% which included 7 novel variants. In about 53% (i.e. 18/34 cases), molecular testing outcome was actionable since 8 of the 18 underwent prenatal diagnosis, as they were either in their early gestation or had planned a pregnancy subsequent to molecular testing, while ten cases were premaritally/prenatally counseled for the families to take informed decisions as they were in the reproductive age.

**Conclusions:**

The report highlights the importance of NGS-based tests even in a low resource setting as it helps patients, families and healthcare providers in reducing the economic, social and emotional burden of these disorders.

## What’s known?

Next-generation sequencing is a tool which has come into mainstream clinical practice in developed nations. Genetic counseling helps clinicians, patients and families understand the ailments and manage it appropriately.

## What’s new?

The article demonstrates utility of next-generation sequencing in Indian population and illustrates the importance in using the test for optimal benefits in clinical management, surveillance, prenatal diagnosis and eventually bringing down morbidity and mortality of musculoskeletal disorders. Genetic counseling and follow-up testing increased diagnostic yield to 79%, the highest in musculoskeletal disorders reported earlier. The manuscript also reports 7 novel variants associated with these disorders.

## Introduction

Disorders involving the musculoskeletal system (DMS) are generalized abnormalities of the muscle and skeletal system often marked by short stature and a spectrum of mobility and orthopedic problems [1]. There are more than 450 disorders that involve the DMS and can be clustered into 40 different groups defined by clinical, molecular, biochemical and/or radiographic criteria. It is estimated that 5% of cases with congenital birth defects belong to the category of skeletal dysplasia [2] and some of them can be diagnosed prenatally by ultrasound [1, 3]. The clinical and genetic heterogeneity of these diseases, with several characteristic overlaps, makes definitive diagnosis difficult for Pediatricians, Orthopaedicians and Radiologists using conventional modalities; hence, molecular diagnostics becomes essential.

DMS predominantly affects the muscle, cartilage or skeletal system and is associated with several genetic syndromes. Patients with these disorders in India report either to pediatric, neurology or orthopedic departments with skeletal abnormalities, altered gait, short stature or joint pain. Disability support in low resource countries including India is abysmally low [4] and families with an affected child face several problems in day-to-day life, if more than one family member is affected then their social, financial and emotional troubles are aggravated [5]. Identifying the type of genetic inheritance followed by appropriate genetic counseling helps families understand the pathology and take informed decisions. Based on pedigree analysis, type of inheritance can be deduced in some cases like autosomal dominant (AD), autosomal recessive (AR) or X-linked dominant (XD) and recessive forms (XR) depending on the location of the associated genes [6]. Confirmation for this again comes after molecular diagnosis.

Since many of the DMS have overlapping features, with heterogeneous symptoms it is clinically difficult to make an accurate diagnosis. In a developing country like India, about 80% of the population has to cater to their own medical needs for consultation, diagnostic tests as well as treatment. A delayed diagnosis would put an additional burden on increased consultations, cross-consultations and repeated diagnostic testing. Even with an absolute clinical diagnosis, reproductive decisions cannot be made, thus NGS-based testing though relatively expensive than other biochemical or radiological test, is a one-time investment in facilitating streamlined syndrome follow-up and reproductive decision making. In the present study, we evaluated the role of NGS in diagnosing patients with musculoskeletal abnormalities referred to the Genetics unit of a tertiary care hospital in South India with comprehensive pre- and posttest genetic counseling to help patients and their families in taking informed healthcare and reproductive decisions.

## Methods

During the period January 2015–August 2019, 70 cases with DMS were referred to the department of Genetics and Molecular Medicine, Kamineni Hospitals, Hyderabad, for genetic testing and counseling. Clinical, social and family history of all the cases was recorded in a well-designed proforma by a genetic counselor after a face-to-face interview with patients or parents/guardians of the affected individuals. Of the total cases, 36 were evaluated with diagnostic modalities like karyotyping, multiple ligation probe amplification assays and microarray, inclusive of cases with a clinical diagnosis of neuromuscular disorders like Duchenne muscular dystrophy and spinal muscular atrophy. The remaining 34 cases were deemed appropriate based on clinical features and family history for carrying out clinical exome with NGS. These included two products of conception (POC), which was abortus material, a case of neonatal death and three couples who were analyzed for carrier status in view of bad obstetric history with radiological evidence of skeletal deformities with or without other congenital anomalies in the fetus. Informed consent was taken from patients/parents/guardians prior to obtaining 2 ml of peripheral blood in EDTA or POC tissue as per the Institutional Ethics Committee of Kamineni Hospitals (Registration #ECR/ 58/Inst/AP/2013) guidelines. This study was carried out in accordance with the recommendations of International Council of Harmonization and Good Clinical Practice. All subjects/families gave written informed consent in accordance with the Declaration of Helsinki.

The samples were outsourced to different commercial laboratories at case per case basis depending upon the cost and turn-around time. Major criteria of choosing the testing laboratory were that they analyzed 300 genes currently recommended for DMS and followed standard protocol [7]. Reporting was done in accordance with the American College of Medical Genetics and Genomics and the Association for Molecular Pathology guidelines [8].

## Results

The age range of cases was 2 days to 56 years and age at onset of symptoms was from in utero to 15 years. Affected individuals were 21 males and 10 females making the male to female ratio 2:1 (65.52% males and 34.48% females). Three couples assessed were not included in the ratio calculation.

Pedigree analysis indicated that of the 34 cases, 15 (44.11%) had positive family history with a first/second degree relative who had same or similar disorder, while 19 cases (55.88%) were sporadic. From the pedigree, we estimated five cases to be AD, of which four were confirmed, while the fifth one was an XD disorder confirmed after NGS testing. Fifteen cases were predicted to have AR inheritance, of which 14 were confirmed by the molecular report, whereas the last case was identified to have a XR condition in a child. Only one pedigree showed an XR which was confirmed after molecular testing. Consanguinity was documented in 15 (44.11%) families suggesting AR inheritance; however, only 12 out of 15 consanguineous cases had a confirmed AR disorder post molecular diagnosis (Tables 1 and 2). AD cases identified were 15, XR were 3, and one family was diagnosed to have XD disorder.

**Table 1:** Genotype–phenotype data of patients diagnosed with DMS

Sr. No	Age/gender	Clinical features	Consanguinity	Family history	Variant (VUS—Variant of uncertain significance)	Zygosity Het: Heterozygous Hom: Homozygous Hemi: Hemizygous	Diagnosis
1.1 Osteogenesis imperfecta							
1.1.1	3/F	Frequent falls, multiple fractures, blue sclera	No	+	Pathogenic *COL1A1* c.3008delC p.Pro1003leufsTer105	Het	Osteogenesis imperfecta I
1.1.2	29/M	Cannot walk, recurrent fractures	No	−	Likely pathogenic *COL1A2* c.1045G > T p.Gly349Cys	Het	Osteogenesis imperfecta III
1.1.3	12/M	Recurrent fractures, osteogenesis imperfecta and blue sclera	No	+	Likely pathogenic *COL1A2* c.838G > Ap.Gly280Ser	Het	Osteogenesis imperfecta I
1.1.4	16 y/M	Developmental delay, post fall- multiple cranial bone fractures, rt facial paralysis also, blue sclerae	No	−	Pathogenic *COL1A1* c.1588G > A p.Gly530Ser	Het	Osteogenesis imperfecta II
1.1.5	12 y/M	Recurrent fractures, dentinogenesis imperfecta, slurred speech, dysmorphism, blue sclera, mild scoliosis, high arched palate	No	−	VUS *COL1A1* c.1861 C > A p.Pro621Thr	Hom	Osteogenesis imperfecta I
1.1.6	5/M	Frequent falls, Multiple fractures, Bluish grey sclera	No	+	rs2075555 Polymorphism *COL1A2* c.804 + 80A > C	Hom	Osteogenesis imperfecta I
1.1.7	32/M	Scoliosis, multiple fractures in childhood, paraplegic	Yes	+	VUS *SERPINF1* c.415G > C p.A139P	Hom	Osteogenesis imperfecta VI
1.1.8	5/M	Recurrent fractures and blue sclera	No	−	NA	NA	Negative
1.2 Osteopetrosis							
1.2.1	25/M	Frequent falls since childhood with multiple limb fractures	Yes	+	Pathogenic *CLCN7* c.856CC/T p.Arg286Trp	Het	Osteopetrosis 2
1.2.2	2/M	Hypotonia, macrocephaly, both humerus bones fractured in neonatal period	No	-	VUS *LRP* c.713C > T p.Thr238Met	Het	Osteopetrosis 1
1.3 Split-hand/foot malformation							
1.3.1	7 year/F	Ectrodactyly	Yes	+	Pathogenic *WNT10B* c.499_500delCT (p.Leu167ValfsTer33)	Hom	Split-hand/foot Malformation-6
1.4 Skeletal dysplasia with in utero presentation							
1.4.1	20 weeks/M POC	Polyhydramnios and musculoskeletal dysplasia	No	+	VUS *MTM1* c.413C > T p.Thr138Met	X-Hemi	Myotubular myopathy
1.4.2	20 weeks/M POC	Skeletal dysplasia in utero	Yes	+	Novel Pathogenic *CUL7* c.867_877del p.Gln289HisfsTer2	Hom	3 M syndrome 1
1.4.3	2 days/F	Microcephaly, small sized cerebellum, Dandy walker malformation oligohydramnios, hypoplasia,	Yes	+	VUS *POMT1* c.123-4C > T Novel VUS *POMT1* C.280 + 7_280 + 9AG	Compound het	Walker–Warburg syndrome
1.4.4	Couple	Pregnancy with increased nuchal translucency, absent nasal bone, pleural effusion, small cardiac size with small left ventricle, bilateral talipes	Yes	+	Novel Pathogenic *CEP290*c.6134C > T, p.Ser2045Leu VUS *CEP290* c.7394_7395del, p.Glu2465ValfsTer2	Het	Joubert syndrome 5
1.4.5	Couple	Microcephaly in fetus	Yes	−	Likely pathogenic *RBBP8* c.604 + 1G > T	Het	Seckel syndrome 2
1.4.6	Couple	Child with skeletal dysplasia in utero with respiratory insufficiency at birth	Yes	−	VUS *COL11A2* c.3133G > G/T p.Gly1045Ter	Het	Fibrochondrogenesis 2
1.5 Short stature							
1.5.1	31 year/F	Short stature, renal rickets	No	+	Pathogenic *PHEX* c.1601C > T p.Pro534Leu	Het	Hypophosphatemic rickets
1.5.2	12 y/F	Atrophy of brain stem, pons and cerebellum, short stature	Yes	+	Pathogenic *ERC66* c.4063-1G > C (3’ Splice site)	Hom	Cockayne syndrome B, Cerebrooculofacioskeletal Syndrome 1
1.5.3	4 year/M	Oligohydramnios, short stature, poor suck, global development delay, microcephaly,	No	−	VUS *CUL4B* c.65G > A p.Gly22Asp	Hemi	Cabezas type of X-linked syndromic mental retardation
1.5.4	5 year/M	Developmental delay, dysmorphism short stature, toe walking	No	−	VUS *AHDC1* c.Gly2618Thr	Het	Xia–Gibbs syndrome
1.6 Joint stiffness and pain							
1.6.1	21/M	Bone pain, Erlenmeyer flask deformity of distal tibia and fibula, thrombocytopenia, renal parenchymal changes, renal cortical cyst, elevated levels of alkaline phosphatase and atrial septal defect	Yes	+	Novel Pathogenic *SLCO2A1* c.325delG p.Ala109LeufsTer64	Hom Het	Primary hypertrophic osteoarthropathy, autosomal recessive 2
1.6.2	16 y/M	Joint restrictions, delayed speech, seizures, breathing problems	No	−	Pathogenic *CCN6:* c.156C > A p.Cys52Ter	Hom	Pseudorheumatoid dysplasia
1.6.3	8 y/F	Difficulty walking, stiffness in left knee, pigeon chest genu valgum, avascular necrosis in right hip	No	−	Pathogenic *COL9A1* c.2755C > T p.Pro919Ser	Het	Multiple Epiphyseal Dysplasia
1.6.4	8 m/M	Joint swellings and hyper pigmentation	Yes	−	Novel Likely pathogenic *ANTXR2* c.54_60dupGCTG TGG (p.Leu21Alafs*26)	Hom	Hyaline fibromatosis Syndrome
1.6.5	30/F	Café au lait spots, scoliosis, back pain	No	+	Pathogenic *NF1* c.4968_4969delAG (p.Asp1657LeufsTer4)	Het	Neurofibromatosis 1

**Table 2:** Genotype–phenotype data of patients with systemic disorders involving the musculoskeletal system

Sr. No	Age/gender	Clinical features	Consanguinity	Family History	Variant	Zygosity	Diagnosis
2.1 Mucopolysaccharidosis							
2.1.1	4 year/M	Distended abdomen, developmental delay	No	−	Pathogenic IDUA c.1882C > T p. Arg628Ter	Hom	MPS I
2.1.2	3 year/M	Coarse facial features, developmental delay, recurrent infections and hernia	No	−	Pathogenic IDUA c.1403-1G > A	Hom	MPS I
2.1.3	2 year/M	Dolicocephaly, Regression of gross motor skills, hepatosplenomegaly, renal rickets, osteopenia, abnormal liver function and loss of subcutaneous fat Fanconi Bickel Syndrome	Yes	-	Novel Likely pathogenic IDS c.1493 G > C p.Arg498Thr	Hemi	MPS II
2.1.4	3.5 year/M	Kyphoscoliosis, short trunk, knock knees, hepatosplenomegaly	Yes	+	Likely pathogenic GALNS c.647 T > C p.Phe216Ser	Hom	MPS IV A
2.1.5	7 year/M	Hypocalcemic seizures, hypoparathyroidism, delayed milestones, hypertelorism, high arched palate, depressed nasal bridge, upturned nose, long philtrum, low set ears, platyspondyly, corneal clouding and sternum protuberance	Yes	+	VUS GUSB c.1499G > T; p.Cys500Phe	Hom	MPSVII
2.2 Mucolipidosis							
2.2.1	7 year/F	Underdeveloped femoral heads, Difficulty in holding things, Unconventional sitting position	Yes	−	Pathogenic GNPTG c.196C > T p.Arg66Ter	Hom	Mucolipidosis-III
2.2.2	4 year/F	Short stature, clubbing of digits, hepatosplenomegaly	No	−	VUS GNPTAB c.A32G p.Tyr11Cys c.3335 + 1G > A	Compound het	Mucolipidosis II
2.2.3	9 year/F	Skeletal problems, short stature, micromelia, pain while walking, breathlessness due to scoliosis and collapsed rib cage	No	−			

### Clinical spectrum of the cases

The cases which were evaluated by NGS were broadly classified into disorders involving musculoskeletal system and storage disorders depending on whether or not they showed systemic involvement.

### 1.Disorders involving musculoskeletal system

Seventy-six per cent (26 of the 34) cases were identified as musculoskeletal disorders with genetic basis after radiological and clinical criteria and were categorized as:

#### Osteogenesis Imperfecta (OI)

Osteogenesis Imperfecta (OI) is a genetic disorder affecting the connective tissue and is mainly characterized by extremely fragile/brittle bones that fracture with trivial traumas [9]. OI is of more than ten types based on clinical features that often overlap. Severity varies with type I being the mildest form and type II being the severest form. COL1A1 and COL1A2 gene mutations are responsible for more than 90% of all cases of OI; however, other genes have also been associated [10].

The eight cases (1.1.1–1.1.8 cases) clinically diagnosed as OI had common features such as recurrent fractures and blue sclera. Their age of disease onset was from in utero to 15 years. Seven of the patients were born of non-consanguineous marriage and four of these had another family member affected (Table 1). Four out of eight cases were identified (50% diagnostic yield) with pathogenic heterozygous sequence variant in COL1A1 or COL1A2 genes indicating AD inheritance. A homozygous intronic variant in COL1A2 gene which is known to be polymorphic and may be associated with phenotype was found in a 5 year old, with childhood onset recurrent fractures, blue sclera and a positive family history. Two cases were identified with homozygous variant of uncertain significance (VUS) in COL1A1 gene and SERPINF1 gene; it is interesting to note that the case 1.1.5 with COL1A1 who had homozygous VUS also exhibited additional clinical features like dentinogenesis imperfecta, slurred speech, dysmorphism and mild scoliosis indicating the severity likely caused due to homozygosity. Segregation analysis may be useful to understand the significance of these variants. However, no significant variant correlating with phenotype was identified in case 1.1.8.

#### Osteopetrosis

Osteopetrosis is a rare genetic disorder characterized by an increase of bone mass due to defective osteoclast function. Patients typically display spontaneous fractures, anemia, and in the most severe forms hepatosplenomegaly and compression of cranial facial nerves leading to deafness and blindness [11]. We confirmed diagnosis of two cases of osteopetrosis (1.2.1–1.2.2). A heterozygous pathogenic variant in CLCN7 gene indicates osteopetrosis type 2 (1.2.1) and the other, a VUS in LRP gene known to cause osteopetrosis type 1 (case 1.2.2).

#### Split-hand/foot malformation-6

Split-hand/split-foot malformation or ectrodactyly is a genetic disorder characterized by the complete or partial absence of some fingers or toes, often combined with clefts in the hands or feet [12]. Two sisters (1.3 case) with ectrodactyly born to consanguineous parents were identified with homozygous pathogenic variant in WNT10B gene confirming the diagnosis of split-hand/foot malformation 6.

#### Skeletal dysplasia with in utero presentation

It is well established that DMS can be identified in the prenatal period. Two POC, one perinatal death and three couple samples were assessed in this category. Couple samples were analyzed because of BOH with skeletal involvement and unavailability of samples from the affected fetus. Analysis of the male fetal sample (case 1.4.1) revealed hemizygous VUS in MTM1 gene diagnosis of XR myotubular myopathy, which is associated with skeletal dysplasia and polyhydramnios in utero, both characteristics, were reported in the ultrasound report available [13]. Since the couple was planning their next child, the mother was evaluated and was identified to have the same variant in heterozygous state confirming carrier status. This also explained the childhood death of two maternal uncles which was documented in the pedigree. Hence, this MTM1c.413C > T p.Thr138Met variant can be considered as a novel pathogenic variant after confirmation with functional analysis.

The second POC sample (case 1.4.2) revealed a novel pathogenic homozygous variant c.867_877del causing protein truncation p.Gln289HisfsTer2 in CUL7 gene causative of 3 M syndrome, which has a phenotype of severe prenatal and postnatal growth retardation, long, slender tubular bones, reduced antero-posterior diameter of the vertebral bodies and delayed bone age [14]. While the sample from a neonate who passed away (case 1.4.3) with a clinical diagnosis of Dandy walker with radiological evidence of malformation and oligohydramnios exhibited two compound heterozygous VUS (which included novel variant c.280 + 7_280 + 9AG) in POMT1 gene causative of Walker–Warburg syndrome [15]. Segregation analysis was recommended in the above-mentioned two cases.

Three couples were screened by NGS in view of consanguinity and BOH with DMS. Two couples were identified to be carriers for AR disorders with single heterozygous causative variant, whereas one couple had different causative variants in same gene contributing to compound heterozygosity in offspring. Case 1.4.4 was identified with compound heterozygosity comprising of a novel pathogenic (c.6134C > T, p.Ser2045Leu) variant and VUS in CEP290 associated with Joubert syndrome 5 (OMIM # 610 188). Case 1.4.5 revealed heterozygosity in both partners for likely pathogenic variant in RBBP8 causative of Seckel Syndrome 2 (OMIM # 606744), whereas case 1.4.6 revealed both parents to be heterozygous carriers for a VUS in COL11A2 associated with fibrochondrogenesis 2 (OMIM # 614524).

#### Short stature

Although short stature is observed in most skeletal dysplasia cases, four of our syndromic cases were found to have short stature in addition to other features. A female member of a family where multiple members had renal rickets was tested and identified to have a pathogenic variant in PHEX gene confirming the diagnosis of X-linked dominant hypophosphatemic rickets [16].

Other three pediatric cases had short stature as a part of a developmental disorders, a homozygous pathogenic variation in ERC66 gene associated with Cockayne syndrome B [17], cerebrooculofacioskeletal syndrome 1 was identified in case 1.5.2, while the third case exhibited VUS in CUL4B gene causative of Cabezas type of X-linked syndromic mental retardation [18] and the fourth case was identified to have heterozygous VUS in AHDC1 associated with Xia–Gibbs syndrome [19].

#### Joint stiffness and pain

Two cases of arthritis as a part of systemic disorders were evaluated in this study. Case 1.6.1 of 21-year-old male with clubbing of digits, Erlenmeyer flask deformity of distal tibia and fibula, thrombocytopenia and bilateral polycystic kidneys was identified to have a novel homozygous pathogenic variant c.325delG causing protein truncation p.Ala109LeufsTer64 in SLCO2A1 gene diagnosing it as primary hypertrophic osteoarthropathy, autosomal recessive 2 (PHOAR2) [20]. It is interesting to note that the same individual was also identified with a heterozygous pathogenic variant in PKD2 gene c.637C > T p.Arg213Ter associated with AD polycystic kidney disease explaining the renal phenotype. Case 1.6.2 was identified with a pathogenic homozygous variant in CCN6 gene causative of autosomal recessive pseudorheumatoid dysplasia manifesting as joint stiffness since the young age of 16 years [21].

A female who had joint restrictions, genu valgum and avascular necrosis was identified to have a pathogenic variant in COL9A1 gene associated with multiple epiphyseal dysplasia [22]. A 8-month-old male born to non-consanguineous parents presented with hyperpigmentation and joint swellings and was reported to have novel likely pathogenic duplication c.54_60dupGCTGTGG causing premature truncation (p.Leu21Alafs*26) in ANTXR2 gene causative of hyaline fibromatosis, a condition where there is deposition of amorphous hyaline material in skin and visceral organs [23]. A 30-year-old female with family history of cancer who has café au lait spots was assessed and found to have a variant in NF1 gene associated with neurofibromatosis [24].

### Storage disorders

Lysosomal storage disorders (LSDs) are a large group of more than 50 different inherited metabolic diseases which, in the great majority of cases, result from the defective function of specific lysosomal enzymes and non-enzymatic lysosomal proteins or non-lysosomal proteins involved in lysosomal biogenesis [25].

#### Mucopolysaccharidoses (MPS)

MPS is characterized to seven subtypes and are caused by deficiencies in the lysosomal enzymes necessary for the degradation of glycosaminoglycans (GAGs). Storage of GAGs affects the skeletal tissue, cartilage and connective tissues, as well as the peripheral and central nervous system [26].

Of the five cases clinically suspected with MPS, three were consanguineous and other two were non-consanguineous. Pathogenic variations in IDUA were identified in two cases diagnosing MPS I. A novel hemizygous likely pathogenic missense variation c.1493 G > C causing protein change p.Arg498Thr was identified in IDS gene in a 2 yr/male patient confirming MPS II. Two brothers with knock knees and hepatosplenomegaly had a likely pathogenic homozygous variant in GALNS gene which is associated with MPS IVA. Another 7-year-old male with intellectual disability, corneal clouding was identified to have a homozygous VUS in GUSB gene causative of MPS VII. The younger brother of the proband was identified with the same variant in GUSB gene followed by enzyme analysis showing deficient β-glucuronidase activity thereby confirming the pathogenicity of the VUS identified.

#### Mucolipidosis

Mucolipidosis has the clinical and biochemical features of both MPSs and sphingolipidoses, being characterized by the accumulation of glycoproteins and glycolipids [26]. In our cohort, there were two clinical cases suspected of mucolipidosis with consanguinity in one case. Pathogenic GNPTG homozygous variant was identified in a 7 year/female. Two VUSs in GNPTAB with compound heterozygosity were identified in another female child of 4 years. A 9-year-old female (2.2.3) born to non-consanguineous couple with storage disorder-like symptoms and elevated urine GAGs was suspected to have mucolipidosis in view of no likely causative variants in MPS genes; however, no diagnostic variant was identified to confirm mucolipidosis in this patient.

## Discussion

Traditionally, healthcare services in India revolve around the specialist clinicians and patients that have direct access to Pediatricians or Orthopaedicians without going through a primary physician. They are responsible for recommending healthcare interventions and for referring patients to the Genetic units or ordering tests. Until recently, DMSs were diagnosed solely on biochemical and radiological findings and were rarely confirmed by specific gene mutation analysis [25]. A publication by Uttarilli et al. [26] described the shift from single-gene testing to panel testing over a decade from multiple centers in India since in most cases large single-gene Sanger testing is more expensive than NGS-based panel testing with a better diagnostic yield.

**Fig. 1:**
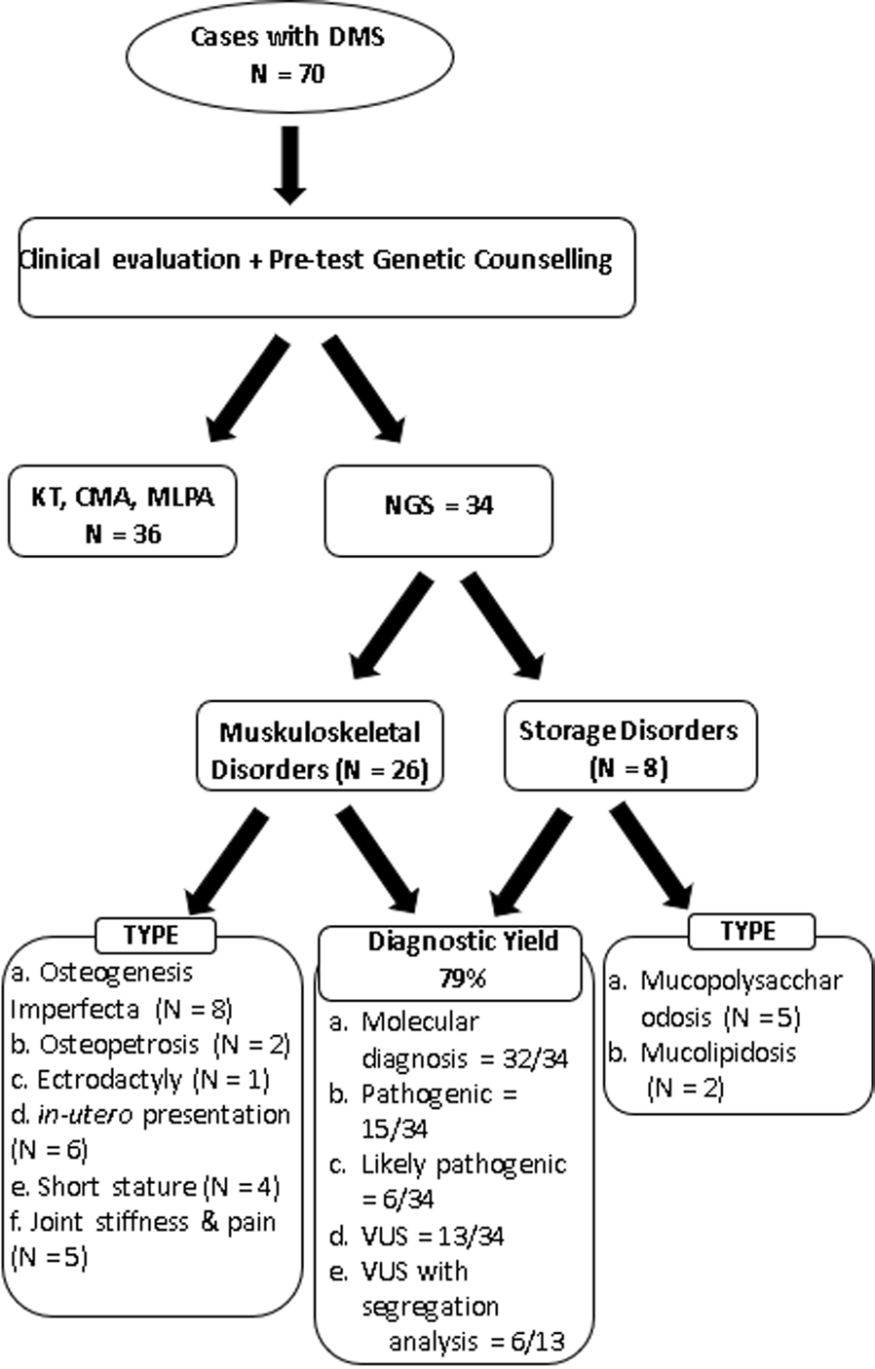
Schematic representation of cases recruitment, outcome of results and diagnostic yield

A total of 94% (32/34) cases were identified with a variation in genes likely causative of the disorder, thereby establishing genotype–phenotype correlation. Of the sequence variants identified, 15 were pathogenic, 6 were likely pathogenic, and 13 were variants of uncertain significance according to ACMG guidelines [27]. Seven novel variants associated with the conditions were identified. Twenty-one pathogenic and likely pathogenic variants were identified and segregation/carrier analysis of 6 out of the 13 VUS was confirmed as pathogenic (cases 1.1.7, 1.4.1, 1.4.3, 1.4.4, 1.5.3, 2.1.5) thus giving a total yield of 79% (27/34) where the patient had definitive molecular diagnosis. Cases 1.1.8 and 2.2.3 were not identified with any clinically significant variants in the gene panel used. Hence, whole exome sequencing may be required for screening a larger panel of genes or identifying novel genes associated with the DMS. Complete diagnostic outcome of case results was represented in the form of schematic diagram (Fig. 1).

As compared to the reports from Manipal [28] and Kerala [29], we observed 44% consanguinity in our cases, against their 37% and 11.6%, respectively. When considering only AR disorders, the rate of consanguinity was seen to be 80% which is significantly higher compared to Manipal data of 43% and 31% from the Kerala publication.

There is a requirement to have a definitive diagnosis for patients and families with genetic diseases to understand the course of the disease and available treatment/ management options to take informed healthcare decisions especially for MPS. Over the past few decades, remarkable advances in molecular diagnosis with the advent of affordable NGS tests make it possible to analyze several genes in a single disease-targeted panel test [30]. A boom in bioinformatics has further helped in interpreting NGS data efficiently especially in countries like India, where databases for variants are not available for their specific population [31]. The data generated will help us develop a disease specific gene panel unique for our population, if all companies donate their raw data files into a common database. This will also help in focusing our research on studying the functional significance of reported VUS showing phenotype correlation and confirming if they are novel pathogenic variants for our population.

The NGS test was helpful in counseling ten premarital/pre-conceptional cases and eight families benefited as they could undergo prenatal diagnosis. Thus, with the current diagnostic yield, 53% of the families could take informed reproductive decisions based on NGS testing who are currently in their reproductive age; the other 26% are equipped with molecular variant information that will facilitate premarital and prenatal counseling and testing in the near future. The limitation of our study is smaller sample size and patients recruited from single center/city. Further we plan to analyze more samples from different units or cities for diagnosis, treatment/management and to plan further prenatal testing.

## Conclusion

This study shows that NGS is an important tool for effective diagnosis of DMS even in a low resource country like India as its diagnostic yield is 79% after a proper pretest and posttest counseling. The patients/families in our country usually consult different hospitals/clinicians in the hope of diagnosis/treatment and undergo repeated tests which disappoint them, many of them in the absence of diagnosis have another child with the same disease, which is devastating. Other members of their family may get affected as no preventative carrier testing was advised or they were not informed about prenatal testing. Also, syndrome-specific management in MPS and similar cases could have been planned in affected individuals. All this can only be facilitated by a pre- and posttest genetic counseling to educate families adequately after a molecular test. This exercise would be instrumental in taking informed reproductive decisions significantly reducing the morbidity and mortality burden on families and society.

## Data Availability

Any additional information can be shared upon request.

## Abbreviations

NGS: Next-generation sequencing
DMS: Diseases associated with musculoskeletal systems
AR: Autosomal recessive
AD: Autosomal dominant
XD: X-linked dominant
XR: X-linked recessive
OI: Osteogenesis imperfecta
LSD: Lysosomal storage disorders
POC: Products of conception
MPS: Mucopolysaccharidoses
VUS: Variant of uncertain significance

## References

[1] Alanay Y, Lachman RS (2011). A review of the principles of radiological assessment of skeletal dysplasias. J Clin Res Pediatr Endocrinol.

[2] Gnoli M, Pedrini E, Mordenti M, Tremosini M, Sangiorgi L (2014). Skeletal dysplasias: approach to the clinical diagnosis and implication of appropriate diagnosis for knowledge and research studies in these rare diseases. Hereditary multiple Osteochondromas as example/paradigm. Italian J Pediatr.

[3] Parilla BV, Leeth EA, Kambich MP, Chilis P, MacGregor SN (2003). Antenatal detection of skeletal dysplasias. J Ultrasound Med.

[4] Ministry of Statistics and Programme Implementation, Government of India Report on persons with disabilities in India. (2018), NSS Report no. 583 (76/26/1)

[5] Golics CJ, Basra MKA, Finlay AY, Salek S (2013). The impact of disease on family members: a critical aspect of medical care. J R Soc Med.

[6] Krakow D (2015). Skeletal dysplasias. Clin Perinatol.

[7] De Groot LJ, Chrousos G, Dungan K, Feingold KR, Grossman A, Hershman JM, Koch C, Korbonits M, McLachlan R, New M, Purnell J. Endotext [Internet]

[8] Zhytnik L, Maasalu K, Reimann E, Prans E, Kõks S, Märtson A (2017). Mutational analysis of COL1A1 and COL1A2 genes among Estonian osteogenesis imperfecta patients. Hum Genomics.

[9] Coudert AE, de Vernejoul MC, Muraca M, Del Fattore A (2015). Osteopetrosis and its relevance for the discovery of new functions associated with the skeleton. Int J Endocrinol.

[10] Van Buggenhout G, Fryns JP (2003) The NORD guide to rare disorders

[11] Amburgey K, Tsuchiya E, de Chastonay S, Glueck M, Alverez R, Nguyen CT, Rutkowski A, Hornyak J, Beggs AH, Dowling JJ (2017). A natural history study of X-linked myotubular myopathy. Neurology.

[12] Huber C, Delezoide AL, Guimiot F, Baumann C, Malan V, Le Merrer M, Da Silva DB, Bonneau D, Chatelain P, Chu C, Clark R (2009). A large-scale mutation search reveals genetic heterogeneity in 3M syndrome. Eur J Hum Genetics.

[13] Huber C, Delezoide AL, Guimiot F, Baumann C, Malan V, Le Merrer M, Da Silva DB, Bonneau D, Chatelain P, Chu C, Clark R (2009). A large-scale mutation search reveals genetic heterogeneity in 3M syndrome. Eur J Hum Genetics.

[14] Ruppe MD. X-linked hypophosphatemia. InGeneReviews®[Internet]; 2017. University of Washington, Seattle.

[15] Wang X, Huang Y, Yan M, Li J, Ding C, Jin H, Fang F, Yang Y, Wu B, Chen D (2017). Molecular spectrum of excision repair cross-complementation group 8 gene defects in Chinese patients with Cockayne syndrome type A. Sci Rep.

[16] Okamoto N, Watanabe M, Naruto T, Matsuda K, Kohmoto T, Saito M, Masuda K, Imoto I (2017). Genome-first approach diagnosed Cabezas syndrome via novel CUL4B mutation detection. Hum Genome Var.

[17] Murdock DR, Jiang Y, Wangler M, Khayat MM, Sabo A, Juusola J, McWalter K, Schatz KS, Gunay-Aygun M, Gibbs RA (2019). Xia-Gibbs syndrome in adulthood: a case report with insight into the natural history of the condition. Mol Case Stud.

[18] Zhang Z, He JW, Fu WZ, Zhang CQ, Zhang ZL (2013). Mutations in the SLCO2A1 gene and primary hypertrophic osteoarthropathy: a clinical and biochemical characterization. J Clin Endocrinol Metab.

[19] Bhavani GS, Shah H, Shukla A, Dalal A, Girisha KM. Progressive pseudorheumatoid dysplasia. In: GeneReviews®[Internet]. 2015. University of Washington, Seattle.

[20] Jackson GC, Mittaz-Crettol L, Taylor JA, Mortier GR, Spranger J, Zabel B, Le Merrer M, Cormier-Daire V, Hall CM, Offiah A, Wright MJ (2012). Pseudoachondroplasia and multiple epiphyseal dysplasia: a 7-year comprehensive analysis of the known disease genes identify novel and recurrent mutations and provides an accurate assessment of their relative contribution. Hum Mutat.

[21] Mantri MD, Pradeep MM, Kalpesh PO, Pranavsinh RJ (2016). Hyaline fibromatosis syndrome: a rare inherited disorder. Indian J Dermatol.

[22] Yap YS, McPherson JR, Ong CK, Rozen SG, Teh BT, Lee AS, Callen DF (2014). The NF1 gene revisited–from bench to bedside. Oncotarget.

[23] Filocamo M, Morrone A (2011). Lysosomal storage disorders: molecular basis and laboratory testing. Hum Genomics.

[24] Marques AR, Saftig P (2019). Lysosomal storage disorders–challenges, concepts and avenues for therapy: beyond rare diseases. J Cell Sci.

[25] Nampoothiri S, Yesodharan D, Sainulabdin G, Narayanan D, Padmanabhan L, Girisha KM, Cathey SS, De Paepe A, Malfait F, Syx D, Hennekam RC (2014). Eight years experience from a skeletal dysplasia referral center in a tertiary hospital in Southern India: a model for the diagnosis and treatment of rare diseases in a developing country. Am J Med Genet A.

[26] Uttarilli A, Shah H, Bhavani GS, Upadhyai P, Shukla A, Girisha KM (2019). Phenotyping and genotyping of skeletal dysplasias: evolution of a center and a decade of experience in India. Bone.

[27] Rehm HL (2013). Disease-targeted sequencing: a cornerstone in the clinic. Nat Rev Genet.

[28] Lelieveld SH, Veltman JA, Gilissen C (2016). Novel bioinformatic developments for exome sequencing. Hum Genet.

[29] McKenna A, Hanna M, Banks E, Sivachenko A, Cibulskis K, Kernytsky A, Garimella K, Altshuler D, Gabriel S, Daly M, DePristo MA (2010). The genome analysis toolkit: a MapReduce framework for analyzing next-generation DNA sequencing data. Genome Res.

[30] Richards S, Aziz N, Bale S, Bick D, Das S, Gastier-Foster J, Grody WW, Hegde M, Lyon E, Spector E, Voelkerding K (2015). Standards and guidelines for the interpretation of sequence variants: a joint consensus recommendation of the American College of Medical Genetics and Genomics and the Association for Molecular Pathology. Genet Med.

[31] https://www.omim.org

